# Guy's Hospital Reports

**Published:** 1839-04

**Authors:** 


### Art. VI.

*Guy's Hospital Reports.* No. VI. and No. VII. *April—October,* 1838. (Vol. III.)—*London.* 8vo. pp. 479.

The sixth Number of this most valuable publication opens with an Introductory Address, by Dr. Barlow, in which he glances with justifiable complacency at the progress which the work has already made in the good opinion of the medical public; and reverts to the fact, highly creditable to the medical school of the great hospital from which it emanates, that no inconsiderable portion of the materials of the publication is furnished by the clinical pupils. The habits of close observation, and the stimulus to methodise and record what has been observed, which naturally result to the students from being thus honorably admitted to participate with their teachers in contributing their labours towards a work of such high practical aims, cannot fail to operate beneficially on their future careers. In this, as in a thousand other instances, we mark with pleasure the vast improvement which is going forward in respect to that which is, after all, the essential business of medical education,—we mean clinical instruction. It is only those who, like ourselves, recollect the general state of knowledge amongst the yearly pupils of some of the great London hospitals some years ago, and the kind of anti-reciprocity feeling which, in too many instances, existed between the teachers and the taught, who can fully appreciate this improvement.

Much, however, as we rejoice at the share which the pupils take in furnishing the rough materials, we trust that the gentlemen who are individually responsible for the form and character of each monograph, will ever, as well from respect to their readers as to themselves, steadily keep in mind the necessity of careful selection and condensation on their part, if they would give to the publication all the value of which it is susceptible. To young practitioners, and indeed to all, of whatever age, whose field of action does not afford the benefit of clinical instruction on a large scale, such a work as the present must prove quite invaluable; nor can we conceive any professional standing so advanced as to justify the reception of a publication, emanating from individuals of such acknowledged ability and experience, with indifference.

A knowledge of the difficulty there is in effecting the cooperation and commanding the continued zealous exertions of several individuals in any joint literary production, made us, we confess, apprehensive as to the possibility of the Guy's Hospital Reports permanently sustaining themselves at the high level they had already reached: the contents of the present and of the immediately preceding Numbers, however, go a long way towards assuaging such fears. May they have successors innumerable of equal merit!

We think we shall best evince our high sense of the importance of this volume by noticing separately each of its numerous articles.

I. *On Spermatocele, or Varicocele of the Spermatic Cord.* By Sir Astley Cooper.—The excellent author prefaces his communication with an account of the causes, symptoms, and diagnosis of varicocele; but, as these have been already published in his valuable works, we are relieved from the necessity of reprinting them. After an exposition of the means of cure usually adopted, he speaks of that lately recommended by Mr. Wormald in the following terms: "It has been advised to draw the scrotum through a ring, and fix it there; but, as it may be readily believed, this has no advantage over the sling support; and is a much greater annoyance to the patient's feelings, either than the disease itself or the bandage which he is called upon to wear." Sir Astley's method (which is new) consists in the removal of a portion of the skin of the scrotum by the knife. The operation is neither painful nor does it excite constitutional irritation. The mode of performing it is as follows:

"The patient being placed in the recumbent posture, the relaxed scrotum is drawn between the fingers; the testis is to be raised to the external ring by an assistant, and then the portion of the scrotum is removed by the knife or knife-scissors; but I prefer the former. Any artery of the scrotum which bleeds is to be tied; and a suture is then made to bring the edges of the diminished scrotum together. The patient should be kept for a few hours in the recumbent posture, to prevent any tendency to bleeding; and then a suspensory bag is to be applied, to press the testis upwards, and to glue the scrotum to the surface. The only difficulty in the operation of removing the scrotum by excision is in ascertaining the proper quantity to be removed; but it adds but little to the pain if a second portion be taken away, if the first does not make sufficient pressure on the spermatic cord. It is of no use to remove a small portion of the scrotum, for, from doing this, I have failed. When the wound has healed, the varicocele is lessened, but not always entirely removed; but the pain and distressing sensations cease, if sufficient of the scrotum be removed. In making the suture in the scrotum, its lower part is to be brought up towards the abdominal ring, to raise and support the testis: as does the suspensory sling when it is worn." (p. 9.)

In one case operated on, the portion of scrotum removed measured four inches in length, and two inches and a half in breadth. This gentleman is now able to ride fifty miles a day without inconvenience; although, before the operation, he could not continue on horseback more than two or three miles.

In another case, Sir Astley's assistant held the scrotum between his fingers, and he removed all that could be easily elevated from the testis and its coverings. " The patient soon gave up the use of the sling support, and lost the pain in the spermatic cord and loins which he had previously sustained."

Five successful operations of this kind have been performed within the last year and a half; and, in order to confirm the value of the improvement, it only remains to be told how long these persons may continue well. Sir Astley's proposition is founded on rational principles: the reparative powers of the scrotum, when half destroyed by gangrene, as in cases of erysipelas, &c. and the effect of the healing process in such cases, in drawing the testicles close up to the abdomen, making thereby a sort of natural suspensory bandage, have often struck us as provisions of nature, the operation of which might be applied with advantage to the improvement of the *surgery* of these parts; and now, that Sir Astley has succeeded, we only wonder at our own stupidity in not having long since thought of so obvious a remedy for the disease in question: but this eminent surgeon has often had the singular felicity of applying at once to practice what others would spend half their lives in thinking about.

In respect of the after-treatment, Sir Astley speaks, perhaps, too lightly. He says, " the patient should be kept for a few hours in the recumbent posture, to prevent any tendency to bleeding." Such, however, are our notions regarding the tendency to œdema of the scrotum from the most trifling inflammatory affections, and the danger of a failure of adhesion consequent thereon, that we are determined, on our first trial of this operation, to confine the patient to bed or the couch until the period of such dangers has passed away.

The concluding observations of the distinguished author should be always borne in mind.

" I wish it," he says, " to be recollected that I only recommend a removal of a portion of the scrotum in those cases of spermatocele in which the patient suffers great local pain; in cases in which he is most urgent to have the swelling and deformity of the part removed; and more especially in those instances in which the function of digestion suffers, and there is a great deal of nervousness and mental depression. For slighter cases a suspensory bandage must be still recommended." (p. 12.)

II. *On Paraplegia depending on Disease of the Ligaments of the Spine.* By Mr. Key.—This may be considered as supplementary to Mr. Earle's and Mr. Stanley's communications on Paralysis in the Medico-Chirurgical Transactions.

How difficult it is often to trace this affection to its true seat during life is acknowledged by all practical men. The object of the present paper is to make known a hitherto neglected source of this disorder,—namely, a local thickening of the posterior vertebral ligament, carried to such a degree as to form a projection into the vertebral canal, and, by making pressure on the spinal marrow, to interfere with the exercise of its functions. The thickened portion of ligament is occasionally much increased in hardness, or even ossified.

Though there are but two cases given, with the appearances on dissection confirmatory of the reality of this cause of paraplegia, they are, we think, quite sufficient to impress on the pathological anatomist the necessity of examining the caliber of the vertebral canal carefully throughout its whole extent in cases characterized, during life, by loss of power in the lower parts of the body; especially in those instances where no distortion nor tenderness on pressure was discoverable.

The first case presented numerous complications: we shall confine our extracts to the morbid appearances observed within the spine.

> "The bodies of the lumbar vertebræ were covered with irregular prominences of bone, and the intervertebral substances projected more than usual within the canal of the spine. The ligaments covering the intervertebral substance between the second and third lumbar vertebræ were found hardened and prominent, and projected so far into the canal as to diminish it by one third of its diameter, thus causing considerable pressure on the spinal marrow." (p. 19.)

The subject of the second case, George Weeks, aged forty-four, tall, and daily exposed by his occupation to more exercise on foot than he was well able to bear, was seized, about twelve months before admission, with a feeling of weakness in the left knee, which soon extended to the foot, accompanied with numbness and a sensation of cold. The right leg became similarly affected in a week or two, and he suffered from catching pains in the hip-joint from motion; but was able to continue his walking employment till within a few days of coming into the hospital. He had for two months back noticed a want of power in the sphincter ani, and had been conscious for about the same period of giddiness, especially upon any exertion. There was now almost entire loss of power in the lower extremities, more particularly the left, with numbness, tingling, and occasional pain, extending from the loins downwards, and inability to empty the bladder except in drops. There was no pain in the back either on pressure or motion, nor weakness in the arms. Appetite good; sexual passion extinct.

The employment of purgatives, together with calomel and cupping on the loins, was productive of no marked benefit. Pain in the hypogastric region began to be complained of, and was soon followed by an abundant and very fetid muco-purulent discharge from the bladder; the urine, which was turbid and fetid, dribbling away incessantly. Extensive sloughing of the nates supervened; and under this complication of evils he speedily sunk.

The appearances on dissection, we are sorry to observe, are given in a very careless and imperfect manner; but for this Mr. Key is not nominally responsible. Thus, with regard to the brain, which was rather small, it is stated that the exterior only was examined! "it seemed natural, but for a little opacity and fluid effusion." "The liver, spleen, and kidneys did not appear healthy." But we are not favoured with the peculiar deviation from the natural condition exhibited by the two former.

"A few points of purulent deposit were found in the kidneys. The bladder and ureters were extensively coated with sloughy, fibrinous, adhesive layers, and their surfaces were bathed in a sanguineous and puriform secretion. The intervertebral substance above the twelfth dorsal vertebra, with the ligaments covering it, presented a slight ridge projecting into the medullary canal, as if an ossification from the edge of one bone tended to unite with a similar growth from the opposite edge. This transverse ridge manifestly narrowed the canal, as was very evident on passing the finger from the wider to the contracted part. On making a vertical section of the spinal column at this part, it appeared that the adjoining edges of bone, as well as the intervertebral substance, projected into the canal; the prominence of the last being the most considerable. The preparation shows, in addition, in the spinal canal a degree of angular flexure forwards at the point of disease. The medulla was very carefully examined, but seemed quite sound." (p. 22.)

The coexistence of urinary disease with paraplegia, enlarged upon in Mr. Stanley's paper, was exemplified in both of Mr. Key's cases, who ascribes the inflammation of the kidneys and neighbouring parts to the depressed condition of the nerves; finding in it a close analogy to the destructive ulceration of the large intestines, so often met with in the latter stages of many protracted disorders. The bony union which was taking place between the adjacent vertebræ, by the ossification of the connecting ligament, reminds us of the unnatural consolidation of the bones of the back so often met with in overworked horses.

Mr. Key suggests that the failure of the lower limbs, so frequently observed in elderly persons of a tall slight make, who have long been in the habit of exercising beyond their strength, (a failure which not unfrequently passes into complete paraplegia,) may occasionally have its source in the morbid alteration just exemplified; especially when no evidence of cerebral disease, and no pain or irregularity in any portion of the spinal column are discoverable. When the muscles of the back prove unequal to its support, during periods of protracted exertion, an unnatural stress will necessarily be thrown on the spinal ligaments, which may readily be conceived to induce morbid alterations in their structure. A rigid adherence to the horizontal posture is the only remedy for such cases.

It is not to be expected, as Mr. K. justly remarks, that the alteration of the ligaments will be uniformly the same, or always attended with precisely the same train of symptoms. Thus, in the young and strumous, the ligaments may be merely thickened and softened, and so cause only a temporary pressure, or they may be so far weakened and yield so as to allow of displacement of the vertebræ, and lead to absorption of the intervertebral substance; or, what is more likely to be the case in persons of advanced age, ossification may take place; and, in either of these latter states, the compression of the cord will most probably be permanent.

It is but rarely, however, that we can expect to have an opportunity of verifying the connexion of paraplegia with disease of the posterior ligament; for either the patient, under judicious treatment, recovers entirely, or, if he fall a victim to the disease, it is generally not until a very advanced period of it, when suppurative ulceration has made such progress as to have obliterated all vestiges of the original commencement of the disease, in the general destruction of bone and ligament.

III. *Researches into the Chemical Nature of Mucous and Purulent Secretions.* By Golding Bird, f.l.s.—This is in many respects an important paper; but we shall have an early opportunity of discussing the subject of it more at length, when we will not fail to notice the author's experiments and views.

IV. *On the Action of Water on Lead, in relation to Medical Police.* By Mr. A. S. Taylor.—It is chiefly to the accurate researches of Dr. Christison that we are indebted for our present greatly augmented knowledge of this subject, and the correction of many serious errors which prevailed in regard to it.

"This gentleman found that pure water, exposed to air, acted with the greatest rapidity on lead; that the chemical change consisted, on these occasions, in the simple formation of a carbonate, which was partly deposited on the lead, but chiefly at the bottom of the vessel; that a minute portion of this carbonate was also, under these circumstances, dissolved; that natural water containing saline matter has little or no action on lead; and that, if any carbonate were formed and dissolved, it was only in the most minute portion, and after the lapse of a considerable interval. He discovered, further, that if certain neutral salts were dissolved in distilled water, they retarded or prevented its corrosive action on lead, allowing the carbonate to deposit itself slowly, and adhere with such firmness to the metal as not to be afterwards removable by moderate agitation; adding, subsequently, to this crust other insoluble salts of lead, the acids of which are derived from the neutral salts in solution; and thus at length forming a permanent and impermeable screen, through which the action of the water cannot any longer be carried on." (pp. 62-3.)

The object of Mr. Taylor's communication is chiefly to confirm the accuracy of Dr. Christison's conclusions by an extensive series of experiments performed at intervals during the last seven years; to determine the best means of preventing water acquiring the poisonous impregnation in question; and to ascertain more accurately, in respect to those neutral salts which are known to exercise a preservative influence, the relative share which the acid and base respectively have therein.

Pure water has no action on lead, if deprived entirely of atmospheric air and of carbonic acid; and, when they are present, the water seems merely to promote the formation of the carbonate by the exertion of a catalytic force, or by holding the gases in solution or in a state favorable to chemical combination. The brighter the metal the more rapidly does the change go forward: hence, *cæteris paribus*, the greater the risk with *new* leaden cisterns, pipes, or roofs, as compared with old ones. It is accelerated likewise when a portion of the lead is out of the water; the carbonate collecting most rapidly on that part which is just above the level of the fluid, and is consequently always humid with the pure water of vaporization. Hence the obvious propriety of keeping such cisterns always at the same level, or at least having them refilled at very short intervals; as the exposure of a large, moist surface to the air for several hours together leads to an extensive formation of the carbonate, to be washed off and diffused through the fluid at its next rising. An augmented temperature likewise facilitates this process, which, accordingly, Mr. Taylor has observed to go on more rapidly in summer than in winter.

The carbonate, which some toxicologists assert, though probably erroneously, to be the only poisonous salt of lead, is soluble, though in a very low degree, in water, which seems capable of taking up about $\frac{1}{5000}$th part of its weight. A much larger additional quantity of it may, however, exist in water in a state of mechanical diffusion, which, together with the sedimentary portion, greatly increases the danger, especially in respect to those culinary operations in which acid substances are employed. Hence the necessity of a precautionary infiltration in all dubious cases is obvious.

River water and spring water, which generally abound in saline substances, have, as compared with rain or distilled water, little or no action on lead; though the very opposite was till lately the prevalent opinion, having been stated by Dr. Lambe and sanctioned by Dr. Thomson. Mr. Taylor has kept the Thames water, with a piece of lead immersed in it, for eight months, without almost any change being produced. Where, however, carbonic acid is present in considerable quantity, the case is much altered. "Whether an unsually large proportion of saline ingredients will give rise to a chemical action between hard water and lead is a point which has not been examined; but it appears to me," says Mr. Taylor, "that this is not improbable, at least, in respect to certain salts."

At the head of substances exercising an influence preventive of the formation of the carbonate stands the sulphate of lime, so common an ingredient of natural waters; the presence of $\frac{1}{4000}$th part of this substance being a sufficient preservative. The other sulphates, and especially that of magnesia, likewise possess this property in a high degree. The muriates are inferior; and still lower in the scale stand the carbonates and nitrates.

Mr. Taylor's experiments confirm those of Dr. Christison as to the beneficial influence depending much more on the acid, and more particularly on the degree of the insolubility of the compound it forms with lead, than on the base. The presence of sulphuric acid in very small quantity in water retards for a time, but does not eventually prohibit, the formation of the carbonate. Phosphate of soda possesses the protective influence in a high degree. Chloride of sodium, or common salt, is but an imperfect preservative: $\frac{1}{4000}$th of it seems to delay the action for a few days; but, within not many weeks, a considerable formation of the chloride and carbonate of lead was found to have taken place. Hence it is obviously unsuitable for those cases where water has to be preserved long in leaden vessels; though, from the facility with which it is obtainable, we may be justified in employing it for shorter periods, as recommended by Dr. Christison, whose experiments have shown that it should not be used in a less proportion than the $\frac{1}{2000}$th of the weight of the water.

As the result of all his investigations, Mr. Taylor comes finally to the conclusion, previously arrived at by Guyton Morveau and Dr. Christison, that the best preservative we can employ, in respect to soft water, is the addition of a small quantity of a solution of sulphate of lime. "But," he continues, "if the water contain much carbonic acid, and more especially if the cistern be close, or if the water containing the gas have to traverse leaden pipes, preservative salts will be of little avail." The introduction of a coil of iron wire he likewise found very effectual in preventing the formation of the poisonous carbonate. The oxide of iron, which is abundantly produced, though it may be in some instances a source of inconvenience, is at least innocuous: hence he suggests the introduction of rings or tubes of sheet-iron within the pipes of lead, when the latter cannot be dispensed with.

Like all Mr. Taylor's writings, this paper is distinguished by ingenuity, clearness of views and of style, and sound judgment.

V. *On the Process of Reparation in simple Fractures of Bones.* By Mr. Bransby Cooper.—This is the continuation of the paper noticed in our Number for October last. The result of the elaborate and judicious series of experiments instituted by Mr. Cooper, in reference to the phenomena of reparation in fractures of bones, almost leads us to the conclusion that, previously to his investigations, we had already realized all the information on that subject which nature deigns to vouchsafe to us; all, in fact, that our senses can well appreciate. Mr. Cooper's investigations are extremely important, as confirming and setting to rights our theoretical notions in respect of this important function of the economy; but we cannot perceive that he has added a single new fact on which the mind can linger. His own summary of the result of his investigations speaks of nothing novel.

"To sum up in a few words the result of the foregoing investigation, as far as it has been carried, I should say that the effects of a simple fracture of bone are, first, the effusion of a greater or less quantity of blood; next, the absorption of its serum and red particles; inflammation of the bone and all the surrounding tissues next takes place: this leads to a deposition of lymph, which soon becomes hardened into cartilages, which, if not different in character, seem, at least, to perform two distinct offices: that secreted by the cellular membrane of the surrounding soft structures produces, by its hardness and contraction, an approximation, or even contact, of its fractured portions; and this, proving a fresh source of excitement to the cartilage secreted by the vessels of the bone, leads to its ossification: whilst that thrown out by the soft parts is, in the end, either absorbed or converted into a structure the same with that which effused it; showing that the vessels of each part are capable of appropriating their blood to the reproduction of the particular structure from which it was derived." (p. 130.)

Mr. Cooper's essay, together with the beautiful and faithful plates which accompany it, should be studied by both practitioner and student.

VI. *On Hemorrhage from the unimpregnated Uterus, associated with Tumours of varying Degrees of Induration and Malignancy.* By S. Ashwell, m.d.—Dr. Ashwell adduces five cases to prove that hard or fibrous tumours of the uterus, by many not regarded as malignant, and by himself viewed as occupying the lowest place in the scale of malignancy, may occasionally give rise to frequent, excessive, and fatal hemorrhage. Such tumours, he states, are observed to grow internally, not imbedding themselves in the walls of the uterus towards its peritoneal covering, but, by their increase of size, stretching and irritating the mucous membrane, and thus giving rise to hemorrhage, with at times a morbid growth of the tissue. That a tumour of this kind may, by commencing just behind the mucous membrane, and increasing inwards, descend even to pass through the os uteri, he admits, but considers it still to be a hard or fibrous tumour, and denies its identity with polypus. He enumerates many points of dissimilarity between this form of growth and polypus. While insensibility is an almost invariable condition of true polypus, this tumour is never bereft of it. The polypus is spongy and copiously permeable by blood; the hard tumour will not admit of injection: and this fact Dr. Ashwell considers as favouring the opinion that the hemorrhage comes, in the latter case, from the membrane covering the tumour, and not from the growth itself. The chief advantage as influencing the treatment, in Dr. A.'s opinion, derivable from the correct diagnosis in this case is, that it will lead to a more careful and protracted management; palliation being all that can be expected. Removal by ligature he considers objectionable, owing principally to the sensibility of the tumour and the difficulty of applying the ligature without including other than the diseased structure. Perfect rest, astringents, anodynes, &c. are the means to be employed. He cautions practitioners against the use of the secale cornutum, which, he says, and we can well conceive it should, has appeared to him to increase the hemorrhage. The cases given fully bear out Dr. Ashwell's views.

VII. *Summary of Cases in the Obstetric Ward, &c.* By Dr. Ashwell.—This summary is interesting, but does not present to our notice anything new. A case of Retroversio Uteri is given, which is so far of importance as showing that the female bladder may be considerably distended, and yet the patient seem to pass a fair portion of urine. We subjoin a brief abstract of the case:—The patient was aged forty-one, had given birth to ten children, and suffered three miscarriages. Three weeks before admission she had been annoyed by constant stillicidium urinæ, followed soon after by increased swelling of the abdomen, with pain in the back and dragging sensation at the umbilicus: still she daily passed a considerable quantity of water, occasionally of a high colour. During the first night after admission into the hospital, she, by voluntary efforts, passed two pints of urine. Shortly afterwards, eleven pints of ammoniacal urine were drawn off by the catheter, and the uterus was then artificially restored to its normal situation. In five days she aborted of a three-months' fœtus, (the bladder having been meantime constantly emptied by the catheter,) and sank in ninety-six hours. Had the true nature of this case been detected at an earlier period, the probability is that the result would have been more favorable. This case carries with it a valuable warning.

As of less importance, we shall pass over two papers by Mr. King, *On White Patches in the Heart*, and *On Compression of the left Bronchus;* as also *A Case of Dislocation of the Femur*, by Dr. Cummins, of Dundee, and *An Account of a large Calculus passed by a young Woman*, by Mr. Harris, of Redruth. Two other papers, by Dr. Bright and Dr. Guy, are continued in the following Number, and will be noticed with its other more important contents, which we shall now take in order.

VIII. *A Contribution to the Pathology of Congenital Deafness.* By

Edward Cock.—In this paper Mr. Cock gives seven cases, in which he examined anatomically the ears of persons deaf from birth; and, for the purpose of rendering the present article more complete, and laying before the reader in one view the whole series of malformations which he has discovered from time to time, he has extracted from the Medico-Chirurgical Transactions the results of his earlier labours. Though in these cases Mr. Cock has not, we think, observed anything of importance not noticed by both earlier and contemporary investigators,—such as Mondini, Ilg, Mürer, Mücke, Bachdalch, and Hyrtl, not to mention Heusinger, Hasselbach, Schallgruber (a most appropriate name for the investigator of the organ of hearing), &c.—still that does not make Mr. C.'s "Contribution" the less acceptable: on the contrary, it makes it the more valuable as confirmatory evidence.

When we dissect the eyeball to discover morbid changes, we think it of importance to examine the state, not only of the sclerotica, cornea, aqueous chambers, iris, and optic nerve, but also the choroid, retina, vitreous humour, and lens. Unfortunately, however, it does not appear to have been considered of equal importance to investigate with care the soft parts contained within the bony shell of the labyrinth: for, notwithstanding we have a good deal recorded on the morbid states of the osseous labyrinth, we may say that we have no observations at all bearing on those of the membranous labyrinth: a strange oversight, certainly, to direct attention to the shell, and neglect the kernel! There are considerable difficulties in the way, no doubt, but they are not insuperable. In museums we see numberless pieces of temporal bones, dry and white like ivory, most ingeniously carved, which, we are informed, are "preparations of the internal ear;" but we almost never see or hear of a real preparation of the internal ear; that is, not only the labyrinthic cavity laid open, but also the membranous labyrinth, with the distribution of the auditory nerve exhibited *in situ.* Some writers on the ear have even confessed, tacitly or openly, they never could see the membranous labyrinth; and, in a "prize essay" on the Ear, recently published, we are told that "the author has several times sought for the calcareous particles of the membranous labyrinth in the human ear, but hitherto unsuccessfully, except perhaps in one instance; [why perhaps?] and, upon enquiry, he cannot ascertain that any of his anatomical friends have seen them, though he is aware that they are now frequently alluded to." The author then goes on to conclude that "*most probably they do not universally exist in the mammalia.*" A most convenient conclusion! All this shows that, as even the healthy structure of the membranous labyrinth is not yet so commonly known as it certainly should be by many of those who write *ex professo* on the subject, we cannot in general expect to have much of any consequence on the morbid states of it.

We shall not follow Mr. C. through his cases, but only glance at the results, as we hope, in an early Number, to consider the whole subject of *Otology* more at large.

Mr. Cock classes the different abnormal appearances he found in his cases under the following heads: 1. An adventitious growth filling up, and thus obliterating, more or less completely, the cavity of the tympanum, enveloping the chain of bones, and encroaching upon the openings of the Eustachian tube, mastoid cells, and fenestra rotunda. 2. Deficiency of the fenestra rotunda; thus rendering one extremity of the labyrinthine canal solid and immovable, instead of yielding. 3. Partial or complete deficiency of the spiral canals of the cochlea. 4. Preternatural enlargement of the aqueductus vestibuli. 5. Deficiency of the semicircular canals. 6. Unnatural solidity or hardness of the temporal bone. In most of the cases detailed several of these appearances existed in combination.

Mr. C. next proceeds to enquire what share these different morbid changes were likely to have as causes of deafness, and comes to the conclusion (we think very justly) that, in cases of the first two sorts, the appearances were quite enough to account for the deafness; though he cautiously abstains from pronouncing whether the appreciable changes in cases of the first sort might or might not have been a mere subsequent formation, depending upon and accruing from a prior defect in the sense of hearing, from some unknown cause.

> "Much remains to be discovered before the causes of congenital deafness can be satisfactorily explained; for I feel assured that in some, if not in many instances, the deviations I have noticed are quite insufficient to account for a total privation of the sense of hearing; and the fact of several cases having occurred in which no malformation could be detected proves that there must be some other defect, either organic or functional, the demonstration or explanation of which has, as yet, eluded our research." (p. 306.)

The great practical question to settle is, "Are any cases of congenital deafness remediable?"—that is, "Can serviceable hearing be restored?" The question is not, "Can the congenitally deaf be taught to speak?" This has been already answered in the affirmative. In our purposed article, we shall examine into Deleau's reputed success in regard to the first question.

Mr. Cock concludes his very sensible and useful paper with the following reflections, which we most heartily reecho:

> "There can be no doubt that, even in cases of congenital deaf-dumbness, the derangement of the organ, whether functional or organic, may exist in different degrees of intensity; that in some instances the mischief consists merely of an imperfection or dulness of hearing, which, from neglect, has entailed the additional misfortune of dumbness on the sufferer; whilst in others it amounts to a total and irremediable privation of the sense. The power of discrimination can only be acquired by the intelligent and scientific aurist, and must be the result of much experience and patient investigation; but the knowledge thus acquired would frequently prove the means of rescuing the hopelessly deaf and dumb from the constitutional injury and bodily suffering consequent upon tedious and painful courses of treatment, indiscriminately adopted by the ignorant and pretending empiric. It would, in many cases, suggest to the upright and humane practitioner the more eligible plan of attending to the cultivation of the other senses through which the mind of man holds intercourse with the external world; and devoting to the mental improvement and education of the sufferer that time and attention, now too often wasted in the employment of means which only serve to impair the mental and bodily powers, while they impose on the credulity of the patient or his friends." (p. 308.)

IX. *Observations on Intussusception as it occurs in Infants.* By Mr. GORHAM.—The principal object of this paper is to point out a particular symptom as confirming the diagnosis of intussusception in infants. This symptom is the discharge of a greater or lesser portion of pure blood by stool, unmixed with any portion of fecal matter, and generally without even the slime or mucus of the intestines. Mr. Gorham believes that, if discharge of pure blood *per anum* takes place in conjunction with constipation and vomiting, it is a symptom pointing out the existence of inflammatory intussusception. He adduces nine cases in support of his opinion; one from his own practice, and some others collected from different sources; in all which cases this symptom was present, occurring early in the disease, and generally coming on immediately after vomiting. Mr. G. considers intussusception under two heads: 1st, Intussusception unattended with inflammation; and, 2, Inflammatory intussusception. He considers the first of these as an extremely common occurrence in infants, not to be recognized during life, but frequently detected on examination of the bodies of infants who have died from other diseases: and here the experience of M. Billard, (*Traité des Maladies des Enfans Nouveaux-nés et a la Mammelle*,) supports that of Mr. Gorham. The second kind of intussusception appears to us rather as an occasional consequence of the first than as an affection *sui generis*. The first kind of invagination reveals itself by no symptoms; but, if it continue beyond a certain period, and offer any obstacle to the passage of food or fecal matters, pain and inflammation are established, and the disease passes to the author's second form.

Mr. Gorham does not attempt in his paper to add anything to the present state of our knowledge on the treatment of this affection. He seems to think highly of the treatment by inflation, and recommends, when all other remedies have failed, "the introduction of the nozzle of a common pair of bellows into the rectum, and gradually inflating the intestine." Three cases are quoted in which this plan of treatment was successfully employed. Injections of large quantities of warm thin gruel are also advisable, used by means of a conical clyster-pipe, to prevent the return of the enema. Two cases treated in this manner, by Mr. Finch, of Greenwich, terminated favorably. The matter of this essay is valuable: there are, however, some gross inaccuracies of style in its composition, which we hope to see amended when Mr. Gorham again appears before the public.

X. *Cases and observations in Medical Jurisprudence.* By Mr. Alfred S. Taylor.—The first is "a case of poisoning by oxalic acid." In this instance the patient, a man aged fifty-six, swallowed seven drachms of oxalic acid, mixed in some warm water, to which he added a glass of rum; the poison was taken an hour after breakfast, which the patient ate in the usual manner. Violent pain and vomiting came on in five or six minutes; to this succeeded cold clammy perspirations, convulsions, and death at the end of a quarter of an hour or twenty minutes, from the period of taking the poison: no treatment was employed. The body was examined seventy-two hours after death. The appearances found in subjects poisoned by oxalic acid present considerable variety. It might, a priori, be supposed that the presence of so violent an irritant would produce, and leave behind in the stomach evidences of the most intense inflammation; such however, in all instances, does not appear to be the case. In the present instance, as well as in one recorded by Dr. Christison, and in some experiments upon animals made by Mr. Taylor, no effects of inflammation were to be perceived; in the case quoted by Dr. Christison from the London Medical Repository, "no morbid appearance whatever was to be seen in any part of the alimentary canal." In Mr. Taylor's case, "the mucous membrane appeared pale and softened, entirely free from rugæ. There were no traces of inflammation or abrasion in any part; and the surface of the membrane presented, throughout, the same characters. The paleness and softening of the membrane were such as we might suppose it to assume after having been boiled some time in water." The mucous membrane of the thoracic portion of the œsophagus was likewise pale, in some places abraded, containing, as did the stomach, a dark brown gelatinous liquid. This liquid is constantly present in the stomach in cases of poisoning by oxalic acid, and results from the mixture of a considerable portion of blood with the poisonous solution taken. In cases of ulceration of the stomach also, where oozing of blood is slowly going on, this dark brown gelatinous liquid is commonly present, and results, according to the researches of M. Cruveilhier, from the admixture of blood with the free acids of the gastric juice.

The small intestines were slightly inflamed, and contained some dark coloured viscid mucus. The parenchymatous organs,—the lungs, liver, spleen, and kidneys,—were highly congested with dark coloured blood. "The blood throughout the cavities, except in the minute vessels of the œsophagus and stomach, was universally fluid and dark coloured." No mention of the condition of the brain and spinal cord is made: probably nothing morbid would have been found; although in such a case as the present, where the mere local effects of the poison are so slight and unsatisfactory, the fatal issue appears to be made through the medium of the blood upon the centres of the nervous system.

The second communication of Mr. Taylor is a "case of survivorship after extensive rupture of the diaphragm."

Extensive ruptures of the diaphragm are generally fatal, and if not immediately so, commonly leave behind them a series of symptoms by which their existence may be strongly suspected, if not pretty clearly known. A case rarely occurs like the one detailed by Mr. Taylor, in which even the existence of such an occurrence was unsuspected during life; yet, on examination after death, the diaphragm is found extensively torn, and a considerable part of the viscera of the abdomen actually in the cavity of the chest.

The subject of the present case, a sailor aged forty, fell on the deck of his vessel nine months previous to his death, by which fall his ankle was severely injured, and several ribs fractured. The state of the ankle became subsequently so bad, that amputation of the leg was performed by Mr. Morgan. From this he sunk, and died about a month after the operation. The following appearances presented themselves on examination of the body.

"The left lung, which was much contracted, lay superiorly and behind. Its upper portion was pale, sloughy, and crepitant: its middle portion, unusually divided from the rest, pale, soft, and completely hepatized. Inferiorly, this organ was of a dark red colour, empty and compressed. The cause of this abnormal condition now became obvious. Two thirds of this side, or nearly one half of the whole cavity of the chest, was filled up by the distended stomach and a long curve of the arch of the colon. These parts, which had protruded through an aperture in the diaphragm, were covered with a great deal of thin omentum. It was found, on further examination, that the opening, which was two and a half inches in extent, was situated in the muscular part of the diaphragm, anteriorly and a little to the left of the œsophageal opening. The margin of the aperture, to which the omentum was, in one or two places strongly adherent, was opaque, yellowish, firm, and even. The ascending colon ran on the right side, and a little posteriorly; whilst the descending colon ran out anteriorly. The bowel, in a contracted state, to the extent of about twelve inches, was folded vertically on the right end of the stomach, which was much distended with air; and covered externally in great part by omentum." (p. 367.)

Mr. Taylor next records two cases of open foramen ovale; one in a woman aged fifty, and another in a boy aged eleven. In the first case the patient died from disease of the womb, no symptom of any kind existing from which this condition could be suspected during life. In the second instance some slight blueness of the lips was present a few days before death, and the boy died very suddenly after placing his hand upon his heart and crying out.

XI. *Some Observations on the causes of Strangulation in Hernia, and on the causes of Death: and also on the Rules of Treatment which such considerations enforce.* By J. W. King.—Mr. King tells us that "most herniæ exist for years before they become subject to dangerous strangulation;" in support of this proposition he gives a table of one hundred cases of strangulated bowel, which bear him out in its correctness. On the causes of strangulation in old herniæ, Mr. King appears to entertain some most original ideas. It is not, we are told, owing to the neck of the sac becoming narrower with increasing years. "Certainly not. The hernia increases with time," and the sac with its neck becomes enlarged, in proportion to the size of the protrusion. As long as the general health continues good, an incarcerated bowel or epiploon "resists alike constriction and turgescence." "But with a certain decline of vigour and health, and most commonly with manifest deterioration of the great depurative organs of the body, the case is altered, its own turgescence seems to strangle it, and all the products of inflammation are of a deleterious or fatal kind: its course is happily slow; but a genuine organizable fibrin, a natural ulcerative absorption, or even fair pus, are sought in vain: a tendency to decomposition is the only uniform character; delayed, however, in proportion to the wideness of the sac's mouth."

In our anxiety to lay before our readers all that is valuable in the books and papers submitted to us, we have read and reread the passage just quoted, which professes to explain Mr. King's views on the causes of incarceration in old herniæ; but we are sorry to say without success. To us his language is truly a Gordian knot; we cannot untie it.

The remainder of his communications is occupied by a consideration of the fatal terminations of incarcerated herniæ, both after the employment of the taxis and from the reduction by the operation. It is disfigured by the same ambiguity of expression, the same loose and undefined ideas, which characterize the former parts. The author speaks of "*inorganizable effusions*," "*exalted irritation*," "*latent excitement*," and "*organic deterioration*;" and the reader, amid the chaff of many such unmeaning and high-sounding words, attempts in vain to glean even a few grains that may avail him in the hour of practical need.

XII. *Chemical examination of the Liquor Amnii.* By G. O. Rees, m.d.—This fluid has been analysed by Buniva, Vauquelin, Frommhertz, Dr. Vogt of Bern, and also by Dr. Bostock, the results of whose experiments are given in an article "on albuminous fluids," in the Medico-chirurgical Transactions. From some discrepancy in the analysis of these chemists, Dr. Rees was induced to make some fresh experiments, taking especial care that the fluid was obtained pure; noticing, at the same time, the period of pregnancy at which it was examined, and the condition of health of the subject from whom it was taken. The first analysis was made upon the liquor amnii of a healthy woman, seven months and a half advanced in her pregnancy, upon whom premature labour was induced on account of a contracted and deformed pelvis. The following are the results.

Strongly alkaline . . Sp. gravity, 1008·6

Contained in 1000 parts:

| | |
|---|---|
| Water . . . . . . | 983·4 |
| Albumen (traces of fatty matter) . . . . | 5·9 |
| Albuminate of soda, chloride of sodium . . . . | 6·1 |
| Animal extractive, soluble in water and alcohol, urea, chloride of sodium | 4·6 |
| Traces of alkaline sulphate . . . . | |

The "proportional constitution" of the liquor amnii varies in different individuals at the same period of utero-gestation, as a reference to the other analysis of this fluid, given in Dr. Rees's paper, will clearly show. Whilst, however, the relative proportionate composition varies in different individuals at the same period of gestation, the specific gravity remains the same.

XIII. Dr. Rees next presents us with an *Analysis of Diabetic Blood.*—It is well known that in the several forms of diabetes mellitus chemists have hitherto been unsuccessful in detecting the existence of sugar in the blood. Drs. Wollaston, Henry, and others, could not, in their researches, discover any trace of it. Subsequently, however, Mr. M'Gregor, of Glasgow, has shown that sugar is present not only in the blood and urine, but also in several secretions and excretions. Ambrosiani has since been able to separate crystals of sugar from the serum of diabetic blood. In the present communication Dr. Rees favours us with a description of the manner in which he has been enabled to detect this substance in the blood of patients labouring under this disease. The following analysis is the result of his investigations:

| | |
|---|---|
| Water . . . . . . | 908·50 |
| Albumen (yielding traces of phosphate of lime and iron, on incineration, | 80·35 |
| Fatty matter . . . . . . | 0·95 |
| Diabetic sugar . . . . . . | 1·80 |
| Animal extractive, soluble in alcohol, urea . . . . | 2·20 |
| Albuminate of soda . . . . . | 0·80 |
| Alkaline chloride, with traces of phosphate; alkaline carbonate, and traces of sulphate, the results of incineration . . . | 4·40 |
| Loss . . . . . . | 1·00 |

XIV. *Physiological Observations on the Muscles of the Eye.* By Mr. B. Cooper.—This paper is carelessly written, but contains some novelty. Mr. Cooper divided the superior and oblique muscles in the eyes of several living rabbits, and makes the following deductions from these experiments; "that the oblique muscles, when acting together, suspend the eyeball in a central position in the *orbiter* cavity, moderate the retracting influence of the four straight muscles, and, when acting in succession, without being restricted by the influence of the recti, they roll the eye on its own axis, drawing the globe forward, and at the same time tending, in a great degree, to extend the sphere of vision."

XV. *On the Effect produced on the Pulse by Change of Posture.* By W. A. Guy, M.B. Cantab.—That the pulse is usually higher in the standing posture than when sitting, and in this again than when lying, was made known to the profession by Dr. Bryan Robinson about a century ago. This subject, or *the differential pulse*, as it is called, was revived about fifty years since by Dr. Macdonnell, of Belfast, and touched upon soon after by Dr. Falconer, of Bath.

Dr. Knox, in a paper published in the Edinburgh Medical and Surgical Journal, in 1815, and lately republished in an enlarged form, which we noticed in our Seventh Number, showed that the difference in the pulse alluded to is at its maximum in the morning, and decreases considerably as the day advances. Dr. Graves ascertained that it becomes more marked as the pulse augments in frequency. A number of observations were made by Dr. Hohl, of Halle, on the pulses of pregnant women, and are related in his work on the exploration of pregnancy. They afford the following averages: Pulse, when standing, 94—sitting, 83—lying, 77; proving that the general rule to which we have alluded is not interfered with by the existence of pregnancy.

Dr. Guy's first paper is founded on one hundred experiments, all made on males; they were commenced in the year 1832. "They were all made on healthy males who had not eaten food for at least two hours previously, and had remained at rest for some time, and ordinarily between noon and two o'clock, P.M., before the person had taken any violent exercise or exposed himself to any other cause of excitement." The age of the persons submitted to experiment ranged from twenty to fifty; the great majority, however, were under thirty, the mean age being a little above twenty-seven. The mean results arrived at by Dr. Guy, omitting fractions were, standing, pulse 79—sitting, 70—lying, 67. Hence the mean difference between standing and sitting was 9; that between sitting and lying 3; and between standing and lying 12.

Some of the extreme cases were, however, very distant from these mean results, as is obvious from the tables given. Thus, the maximum difference between standing and lying amounted to 44 beats, or little less than one half of the total number of pulsations in the erect posture; whilst, in seven cases, this difference was so low as 4, or only $\frac{1}{14}$th of the whole number; in three instances it was no more than 2 beats; in three there was no difference at all; and in five the pulse was actually *higher* when lying than when standing; ditto when sitting than when standing, in three; ditto when lying than when sitting, in eleven: in nineteen instances there was no difference between sitting and lying; and in five none between standing and sitting.

These exceptional cases, however, bear but a small proportion to the whole number on which the law rests. If they be struck out of the tables, the mean number of pulsations in the standing, sitting, and lying posture, appear to be 81, 71, and 66, respectively; that is, the difference between standing and sitting = 10 beats or $\frac{1}{8}$th of the whole; the difference between sitting and lying 5 beats or $\frac{1}{14}$th of the whole; that between standing and lying 15 beats or $\frac{1}{5}$th of the whole.* Hence, if we know what the pulse is in the erect posture, and would calculate what it is probably in the sitting posture, we have only to subtract $\frac{1}{8}$th, or from the lying posture $\frac{1}{5}$th. But it must be remembered that these average proportions hold good only in respect to a pulse in a quiet state, and of moderate quickness (60 to 90); the differences increasing in a greatly augmented ratio when the pulse is accelerated: thus, for example, when the pulse is between 90 and 100, standing, the recumbent pulse will be about $\frac{1}{4}$th less; if between 110 and 130, the recumbent pulse will be about $\frac{1}{3}$d less.

Both Dr. Graves and Dr. Knox have regarded the degrees of effect produced on the pulse by posture as a measure of debility. Whether it is a strictly appropriate one must be doubted, when we recollect that the casually accelerated pulse of a stout and healthy individual manifests equal amounts of variation, under the influence of posture, with those observed in the case of an equivalent pulse in disease. The pulse, as it has been correctly stated by Robinson and Graves, is strongest in the horizontal position, so that its maximum of strength and minimum frequency are attained together. It is deduced by Dr. Guy from his own tables, that the exceptional cases, that is, those in which change of posture does not produce its ordinary effect, diminish in frequency as the pulse becomes more rapid. Thus, for example, out of fifty cases, where the pulse was between 51 and 70, there were eighteen exceptions; whilst out of other fifty, where the pulse was between 110 and 130, only one exception occurred. So likewise irregularities of pulse often disappear under the influence of feverish excitement.

By means of a couch revolving on an axis, Dr. Guy satisfied himself of the correctness of Dr. Macdonnell's and Dr. Graves's observations, as to the rise in the pulse being altogether independent of the effort by which the posture is changed. He has moreover proved experimentally what had been only surmised by others before him, namely, that the true cause of the increased frequency of the pulse in the sitting and standing posture, as compared with the recumbent, is the muscular exertion employed in their maintenance. Thus he found that the usual effect on the pulse in the sitting posture was almost entirely superseded when the back was supported, as was likewise the case in the erect posture when the individual leaned upon some high object. Whilst, on the other hand, the pulse in the horizontal posture rose no less than 14 beats on an average, on the body being supported merely under the head and shoulders and under the heels; whilst, in the sitting posture likewise, the circulation was considerably accelerated when the legs

* The difference between the number of pulsations when standing and when lying amounts, according to Robinson, to 14; Macdonnell, 12 to 16; Falconer, $6\frac{3}{4}$; Knox, 10; Graves, 6 to 15; Nick, 15 to 20; Hohl, (in pregnancy) 17.

The large number of experiments on which Dr. Guy's averages, given above, rest entitle them to much consideration.

were raised by a muscular effort to right angles with the unsupported trunk.

Those who, like Blackley, have ascribed the effect on the pulse to the altered position of the heart and its valves in the erect, as compared with the recumbent posture, or, like Arnott, to the change in respect to the gravitation of the blood, seem to have forgotten that, whilst the difference between the pulse of the erect and sitting posture is nearly double of that between the sitting and recumbent posture; yet the above conditions in respect to the heart's gravity remain, whether sitting or standing, nearly the same.

In Dr. Guy's second paper, the subjects of the experiments are females; and he here limits himself to the examination of fifty subjects, instead, as in the former instance, of one hundred; since, "for obvious reasons, opportunities of making correct observations on the pulses of healthy females occur but rarely." The ages of the persons examined in these experiments ranged from eighteen to eighty, giving a mean age of 27·18, and affording the following results: Standing posture, 89·26; sitting ditto, 81·98; lying, 80·24. These numbers give, as a general rule, an average difference of frequency in pulsation between the standing and sitting postures of 7·28; between the sitting and lying, of 1·74; and between the standing and lying, of 9·02. There occurred in these experiments, as in those upon males, many exceptions to the general rule of the pulse being less frequent in the recumbent, less so in the sitting, and least so in the standing posture; with this addition, that the exceptions were more numerous in the female than in the male. The first twenty-seven experiments, however, refer to the general rule, and from them all exceptions are excluded: these give, as the mean age, 26·45; as the average of frequency, in the standing posture, 91·33; in the sitting, 84·37; in the recumbent, 79·74.

The exceptions to the general rule are very numerous in the female, and amount, in their several varieties, to as much, on the average, as 46 per cent.

Dr. Guy next proceeds to the examination of the effect of posture on the pulse of pregnant women, and compares them with those of Professor Hohl: there is some little difference of result from these two examinations; but we are not told, in Hohl's experiments, of all the circumstances under which the examinations were made, neither are the ages of the patients mentioned. In Dr. Guy's experiments, the mean age of the persons examined was 29; the frequency of pulse, in the standing posture, 87; in the sitting, 83; in the lying, 80; the differences, 4, 3, and 7. The difference observed in the comparative frequency of the pulse in different postures is greater as the pulse is naturally more frequent. "The pulse of the adult female exceeds in frequency the pulse of the adult male, of the same mean age, by from 10 to 14 beats. In the erect posture, it is more frequent by about $\frac{1}{8}$th; in the sitting posture, by about $\frac{1}{6}$th; and in the recumbent posture, by more than $\frac{1}{5}$th: but, although the pulse of the adult female is more frequent than the pulse of the adult male of the same mean age, the effect of a change from the erect to the recumbent posture in the male is greater than the effect of the same change in the female, by more than $\frac{1}{3}$d."

The influence of posture on the comparative frequency of the pulse is much modified by age. From an examination of the tables referring to this point, it appears that the influence of posture upon the pulse is less in early youth than adult age, and the modifying influence of age is greater in the female than the male.

Dr. Knox, in the paper before referred to, and Dr. Nick* state that the effect of posture on the pulse is much greater in the morning than in the evening; the results of their experiments are both confirmed by those of Dr. Guy. Thus, in the morning, the mere change from the horizontal to the erect posture increased the pulse by about 15 or 20 beats. The results of seven experiments by Dr. Knox gave, as a mean result, a difference of 29 between standing and lying in the morning, and of 21 in the evening. The results of twenty experiments instituted by Dr. Guy gave, as a mean difference, immediately on getting out of bed, of 10 beats in a minute between the recumbent and the standing postures; after dressing, of 7·70 ; and, immediately before going to bed, of 7·25. These experiments prove that the effect of change of posture is greatest in the forenoon, and least in the afternoon: they likewise establish the fact, first promulgated by Dr. Knox, "that the pulse is less frequent in the evening than the morning. This difference amounts to 11 beats."

The remainder of Dr. Guy's paper is occupied by a comparison between the result of his observations and those of Dr. Graves, made with the revolving board. Here, in the main, they agree: there is some little difference of opinion as to the physiological cause of the difference of frequency of pulse during recumbency and in the erect posture; but this appears to us of little moment. The last experiments were made with the revolving board to ascertain the extent to which the pulse would fall below that of the horizontal posture, by depressing or lowering the body to angles varying between thirty and forty-five degrees; from which Dr. Guy concludes that the natural tendency of the inverted position of the body is to diminish the frequency of the pulse. When the head and body were inverted thirty degrees, the average depression of pulse was $6\frac{1}{2}$ beats; at forty-five degrees, $1\frac{1}{2}$; completely inverted, 7 beats.

These experiments appear to have been performed with great care, and due attention to all circumstances which might have interfered with a correct result. They are physiologically curious; and Dr. Guy deserves the thanks of the profession for the care and assiduity evinced in their institution and performance. We refer the reader to the original paper for a series of tables containing much curious information in reference to the whole subject.

We are pleased to find that the author is engaged in collecting materials for discussing the practical application of the results obtained; and trust that he will at the same time take occasion to elucidate the causes of the numerous exceptions to the general rule, as well as to show the modifying influence exercised by disease of different organs, and more especially of the heart and lungs, both in respect to the rules and the exceptions.

* Beobachtungen über die Bedingungen unter denen die Häufigkeit des Pulses im gesunden Zustand verändert wird. Von G. H. Nick.—Tubingen, 1826. p. 41.

XVI. *Observations on Abdominal Tumours and Intumescence.* By R. Bright, m.d. f.r.s.—In noticing the Fifth Part of these Reports, in our last volume, we announced our intention of postponing the review of Dr. Bright's paper, therein commenced, until its completion. We know not that it is now completed; still we cannot allow longer time to pass without making our readers acquainted with its valuable contents.

The first part of Dr. Bright's paper relates exclusively to the "Acephalocyst Hydatid," and is illustrated by fifteen cases of the disease, the detail of which furnishes very nearly a complete account of the history, pathology, and termination of this affection. Before entering immediately upon the subject of abdominal tumours, Dr. Bright devotes a few pages to the consideration of the regional anatomy of the abdomen. In this we do not find any addition of consequence to what has already been said on this subject by Velpeau and others; still this introduction to the study of abdominal tumours is exceedingly appropriate. It must be recollected that the regional anatomy of the abdomen differs materially from that of the chest. In the latter the organs are fixed, or nearly so, and in a state of health deviate little in their correspondence with certain regions of the external surface of the body: in the former, on the contrary, nothing is precisely definite; the nature of the viscera, and their functions, render them constantly liable to changes of situation; and hence Dr. Bright judiciously observes, "that they are subject to variations from anomalous formations, from changes as to form and extent, according to the state and progress of the operations in which they are destined to assist; we must, of course, be prepared to appreciate and make allowance for such changes when investigating the condition of the abdomen, or speaking of the natural contents of its different artificial divisions."

A plan of the male and female abdomen is given in illustration of this part of the subject, with some ingenious directions for mapping the situation of tumours, and their connexion with or relation to the various organs of the belly.

Dr. Bright prefaces the narration of his cases by a few remarks on the diagnosis of the acephalocyst hydatid tumour of the abdomen. From the variety of situation which such tumours may occupy, the symptoms by which they are accompanied are liable to much variation. This arises from the nature of the organs with which they are connected, or which, by their bulk, they compress. The acephalocyst, in the first instance, is merely characterized by swelling, gradually increasing; an elastic feel, or sense of fluctuation, when examined by the touch; a smooth or tuberose surface; and not, at the commencement, much derangement of the general health. Dr. Bright mentions a case of encysted cerebriform disease, which was confounded with the acephalocyst tumour in this instance: the more rapid progress of the affection, the greater constitutional disturbance, the general irritability of the system, the sallow and unhealthy complexion of malignant disease, constituted the distinguishing differences between the two affections; although the indications afforded by appearance and manual examination were similar. Cruveilhier, in his excellent article "Acephalocyste," in the "Dict. de Médecine et de Chirurgie pratique," adds nothing to the diagnosis given by Dr. Bright.

As the present communication of Dr. Bright is strictly clinical, we shall endeavour to seize upon those facts of practical utility which the histories of the cases appear more particularly to enforce. The acephalocyst hydatid may become a source of fatal disease in the following ways: 1. By its immense bulk pressing upon and impeding the functions of one or more organs, and thus giving rise to a continual constitutional disturbance, under which the patient gradually emaciates, and dies. This is well exemplified in the first and second cases of Dr. Bright. In the first the tumour, by compressing the stomach and intestines, had caused the absorption of the kidneys, which were reduced to mere membraneous sacs, whilst a portion of the tumour was imbedded in the substance of the liver. In the second case, the liver was forced upwards, the chest compressed to a considerable extent, and the respiration considerably impeded. The small intestines were forced into the left iliac region; the stomach partially compressed; the ureter distended from pressure, and the kidney partially absorbed. 2. The presence of the acephalocyst hydatid may become a cause of death, by exciting a fatal peritonitis. This position is exemplified by the detail of the third case. 3. The fatal issue may be caused by the inflammation and suppuration of the parietes of the hydatid cyst itself. The fourth case of Dr. Bright is an example of this mode of termination. 4. Sudden rupture of the cyst is commonly, though not always, followed by sudden death. The eleventh and twelfth cases are illustrative of this mode of termination. Such are the chief modes in which the acephalocyst gives rise to a fatal termination: we shall presently see the circumstances under which its issue is favorable. Occasionally the situation of the hydatid gives rise to very peculiar and obscure symptoms; of this Dr. Bright brings forward a very singular example. In this case a large acephalocyst hydatid was developed in the pubic region, and attached to the posterior part of the fundus of the bladder, pressing so much forward as to prevent entirely the bladder being filled with urine. This was at once the source of the tumour above the pubes, which was taken for a distended bladder, and explained also the constant escape of the urine, and the reason why none, or very little, followed the introduction of the catheter. The pressure of this tumour upon the ureters had occasioned their great distention by urine, and consequently a degree of pressure upon the kidneys, which had produced a very extensive absorption of the substance of both.

"In cases of so doubtful a kind, we might derive diagnostic marks from the history of the tumour, if the patient had sufficient intelligence to assist us in the enquiry: so likewise from the feel of it, which would probably be harder or less regular than of the bladder; but on this no perfect reliance could be placed. The character of the urine which is drawn off would be almost sufficient to decide the question; for, if this fluid be retained any time in the bladder, it generally acquires a much darker hue: and all its sensible properties at once bespeak that it has been long secreted, and concentrated or altered by retention."

The spontaneous favorable terminations of the acephalocyst tumour are three: 1. The cyst may be ruptured from accident or from internal causes, the fluid carried away by the absorbents, and the disease return no more. The thirteenth case illustrates this. 2. The cyst may burst externally, the fluid be discharged, and a perfect cure be the result.

Dr. Bright certainly does not record a case of this character: the only one he gives of spontaneous external rupture (Case xv.) terminated fatally by hemorrhage from an hepatic vein. Plater, Guattani, Roux, and Cruveilhier have, however, related cases of the favorable termination of hydatid cysts, spontaneously opening externally. 3. The cysts may open into the kidneys, the stomach, or intestines, and be voided by expectoration or by stool. Of these modes of termination two cases are recorded by Dr. Bright; one in which an acephalocyst hydatid tumour of the liver opened into the lungs, and was voided by expectoration; and a second, (Case xiv.) in which the cysts passed off by the intestines. Many examples of these modes of termination are recorded, more particularly by Rudolphi;* Collet, Merat, and Andral have also collected the most remarkable examples of these terminations on record.

In regard to the treatment of this class of affections in a medical point of view, Dr. Bright offers us little or nothing. He proposes the internal use of turpentine, electricity, and the local application of ice; but mentions these remedies as subjects merely of "fair speculation," as "unsupported conjectures." We have certainly little faith in the medical treatment of the acephalocyst, and the records of medical science furnish us with no data whatever in reference to this subject. Surgery is a little more promising. Dr. Bright brings forward two cases in which the tumours were emptied by paracentesis: one terminated fatally (Case ix.); the other (Case x.) was followed by success. We must note here, however, particularly, that in the fatal case the discharge was purulent, marking inflammation and suppuration of the parietes of the hydatid cyst; whilst, in the successful case, the fluid was perfectly limpid.

The treatment by opening the tumour by incision or puncture is one which has generally been condemned by all surgical writers, particularly by Lassus, (*Journal de Médecine,* tom. i. p. 135;) and the older writers are full of fatal issues to such operations. Cruveilhier's essay contains five fatal cases of puncture: he mentions, however, some cases which have terminated favorably, but thinks that many of the cases which have ended well have been serous cysts, and not the genuine acephalocyst hydatid. Lately it has been proposed to puncture the cyst by way of exploration, with a needle similar to Dr. Davies's for testing thoracic collections: then, if the contents be perfectly healthy, to open the cyst by means of a caustic issue; to use at first emollient, and then gently stimulating injections, with a view of inducing adhesion or contraction of the walls of the cyst. Récamier has reported several successful cases thus treated by him at the Hôtel Dieu.

In his second communication Dr. Bright passes from the consideration of the acephalocyst hydatid to that of tumours having their origin in the ovary. This paper is, if possible, more valuable than the former: it is longer, and contains a detailed account of twenty-six cases of various forms of ovarian disease. As a contribution to pathology it is of inestimable value, forming, as it does, a complete history of certain forms of ovarian disease. In it Dr. Bright notices four distinct forms of ovarian

* Et procul dubio Hydatidæ ex Hepatis Abscessu in Duodenum penetranti derivandæ. Vol. ii. Par. ii. p. 248.

tumours with fluid contents: 1st. The simple serous cyst. This presents itself as a simple bag, containing serum, whose external surface appears to possess all the attributes of the peritoneum, attached to the surface of the ovary or some neighbouring part, and supplied with blood-vessels from the point whence it arises; sometimes sessile, more frequently attached by a longer or shorter neck; generally single, but occasionally presenting the appearance of being composed of more than one cyst." The simple serous cyst may be congenital; at least, it has been found within a very few moments after birth. It varies in size from a pea to that of an orange. Dr. Bright adduces no case of its having attained to any considerable size, but imagines it may do so: the only cases he gives of this affection (i. and ii.) were discovered after death, in patients dying from other diseases. In one case the age was five months, in the second eighteen years.

The second ovarian tumour consists in a serous distention of the Fallopian tube. Dr. Bright has not observed any instance of this affection sufficiently large to form a distinct elevation above the pubes. Other forms of distention of the Fallopian tube occur in the shape of purulent or scrofulous deposits: these are usually complicated with scrofula or inflammatory affections of the peritoneum.

The third form of tumour, "as a separate disease distinct from others very doubtful," consists "of a simple vesicular body, developed beneath the proper tunic of the ovary; supposed to be produced by an accumulation of fluid in one of the Graafian vesicles." Dr. Bright believes that the large cyst filling the abdomen, which has been sometimes supposed to be the distended Graafian vesicle, is, in fact, the malignant encysted tumour of the ovary. There occurs a condition of the Graafian vesicle, in which "a coagulum more or less stained with blood, or of a somewhat glutinous character, is collected in the vesicle, distending it to the size of a hazel-nut, or sometimes larger." This is not of a malignant character.

The fourth example of ovarian tumour, by far the most frequent, is that which is known commonly under the name of encysted dropsy of the ovary. This tumour Dr. Bright believes to be most frequently of a true malignant character, "essentially a specific disease." In the opinion of Dr. B., this form of tumour originates in the cellular substance of the ovary, involving during its progress the whole substance of the organ, so that it is often quite impossible to say whether it be the meshes of the cellular tissue or of the vesicles of De Graaf which are become the seats of the morbid deposits, or to what extent new structures have been generated." Many of the continental pathologists consider these tumours to originate in the Graafian vesicles.

Passing over the consideration of the symptoms, &c. of the first three forms of tumour, which are not of sufficient importance to detain us, we come to notice the earlier symptoms attendant upon the malignant simple or compound ovarian cyst, as this is a subject of vast importance to the practical physician and surgeon.

"This form of disease seldom shows itself much before the twentieth year, and generally much later; and is not, like the simple cyst, unexpectedly discovered during the examination of children or young persons who have died from other diseases. The first recognized symptom is usually a tumour, not altogether devoid of pain, in one of the inguinal regions; and which, on examination, evidently rises out of one side of the pelvis, and even at this early period is sometimes lobulated or uneven in its form, and unequal in the resistance its different parts afford on pressure. The growth of this tumour is, on some occasions, so unperceived, that, though it may have originated on one side, it has already risen into the pubic and even into the umbilical region; and, when the medical man is first consulted, its lateral origin is with difficulty ascertained. At other times the enlargement is at first slow, and, after some indefinite period of time, the increase takes place suddenly; so that, in a few months, the whole abdomen presents, to a common observer, the size and appearance of pregnancy far advanced." (p. 184.)

Were the histories of diseases clearly and truly detailed to the medical attendant, little comparative difficulty would be experienced in our diagnosis: unhappily, however, this is not the case, and hence considerable embarrassment sometimes arises in forming a correct opinion. Thus, when a gradual enlargement of the abdomen follows the cessation of the catamenia, the case is apt to be mistaken for pregnancy. This happened in the eleventh case detailed by Dr. Bright. Another case is also given of the complication of ovarian disease with pregnancy at page 261. The ovarian cyst may, at certain periods of its history, be confounded with pregnancy; it may simulate or coexist with it. In these instances we must be guided by the history of the disease, the examination per vaginam, and—what Dr. Bright does not mention but which is of vast importance at the period we are called upon to investigate the nature of the affection—auscultation. The ovarian tumour might also be confounded with malignant disease of the fundus of the uterus: from this, the more central situation of the latter will, in some measure, distinguish it. "The hardness of the tumour, and the peculiar abrupt nodules which the diseased uterus presents, contrasts well with the soft and yielding feel which the subsidiary tumours of the compound ovarian cyst usually afford." The ovarian disease might also be confounded, in its incipient state, with a distended or thickened bladder, or an acephalocyst tumour of pelvic origin, as in a case already detailed. In its advanced stages, it might be confounded with hysterical tympanitis. Dr. B. relates a curious case (xxvi.) of a patient labouring under hysterical tympanitis, who was tapped under the supposition that she had ovarian dropsy.

The ovarian tumour, in its earlier stages, commonly presents an uneven surface to the hand: the greater rotundity or projection of one part over another will at this time be apparent. In the advanced stages, the distension of the cyst by fluid gives to the abdomen a rounded form, which contrasts it with the ovoid appearance of ascites. On examination by moderately hard pressure with the hand, a general sense of fluctuation is perceived; interrupted occasionally, if the abdomen be not too tense, by considerable masses of unyielding matter. These, from their situation, might sometimes lead to the supposition that the liver, spleen, or kidneys are involved in the disease: the tumours thus felt, however, are smaller cysts developed in the interior of the parent cyst, giving rise to that kind of tumour which Dr. Bright terms the compound. When the fluid is drawn off from the chief cyst by paracentesis, the more distinct feel of these smaller cysts leaves no doubts as to the character of the disease.

We agree with Dr. Bright that percussion is of vast service in the diagnosis of cases of this description, particularly when the ovarian tumour gradually pushes away the hollow viscera to the opposite side from whence the disease had its origin. "In ascites, the hollow viscera, as long as the peritoneum has undergone no change, float on the surface of the fluid, in whatever position the body may be placed." In ovarian disease, the hollow viscera are constantly on the side opposite to the tumour.

The prognosis in ovarian disease is generally unfavorable: in addition to its generally malignant character, the disease is sometimes combined with scirrhus or tubercular deposits in other organs, as the mammæ, the liver, the glands generally, and the subcutaneous cellular membrane of the abdomen. The bulk of the tumour may so far compress the viscera of the abdomen, or encroach upon the chest, as to render an operation actually necessary. This, a mere palliative measure, may relieve the symptoms for a time: the cyst, however, soon again fills; a second operation is required, and the patient sinks, worn out by the prolonged irritation of the disease, or by accidents to which the operation itself has given rise.

The more frequent accidents that succeed to paracentesis are sudden sinking, peritoneal inflammation, and diseased conditions of the lining membrane of the sac itself. In the latter instance, the mucilaginous fluid, resembling linseed tea, which was drawn off in the first operation, gradually becomes more turbid and offensive, till, in succeeding operations, it is purulent. In examining cases of this description, the lining membrane of the cyst is generally found inflamed and ulcerated. In some instances the cyst is ruptured internally, and a speedily fatal result is the consequence. Again, the cyst may become united by inflammation to the intestines, ulceration be set up, and the contents discharged. Dr. Bright relates a remarkable example of this mode of termination: the discharge took place into the cæcum, and the patient ultimately sunk from intestinal irritation.

Little can be said of the treatment of this formidable disease. Dr. Bright, in our opinion very justly, condemns the attempt at removal by operation. Whilst medical science offers no remedy which can act specifically upon the disease, attention to the general health may do much in retarding its development. Our chief indications are to subdue local and general excessive action, and to maintain the strength. For these purposes "small local bleedings, blisters, and counter-irritation, mild bitters and tonics, the taraxacum and sarsaparilla, the alkalies, and various narcotics," are to be used as circumstances may require.

Dr. Bright concludes his paper with some remarks on the manner and time of performing the operation of paracentesis. This should not be done in the earlier period of the disease; neither should it be delayed till the bulk of the tumour compresses the chest, or injures by its weight any of the organs of the abdomen; of which occurrences Dr. Bright's paper affords many examples. When the tumour has distended the belly to the size of pregnancy, the operation should be resorted to. Before operating, the surface of the tumour should be most carefully examined, in order to ascertain, as far as possible, the part in which the fluid is chiefly accumulated, that we may not push the trocar into a secondary cyst. In one example (Case xv.) the operator passed the trocar into a firm band, formed by the broad ligament of the uterus and the fallopian tube stretched over the tumour. "Very careful manual examination might possibly prevent such an accident." It is sufficient to mention the probability of this, to render the operator extremely watchful against its occurrence. The bladder should be emptied before operation, and the condition of the uterus ascertained per vaginam. The necessity of these precautions must be obvious to all. In conclusion, Dr. Bright recommends all the fluid that can be conveniently abstracted to be taken away; for, above all accidents, he dreads the escape of any into the peritoneum. If we can ascertain whether any adhesion has taken place between the tumour and the abdominal parietes, the point of union is the place for our incision.—This, although not a complete history of ovarian diseases, which Dr. Bright does not profess it to be, is the most valuable monograph on the subject of the encysted dropsy of the ovaries with which we are acquainted.

The third communication of Dr. Bright's is limited to the consideration of diseases of the spleen. It is illustrated by twenty-eight cases of different forms of splenic disease, occurring singly, in their connexion with different morbid states of the economy at large, or with affections of different organs concerned in the digestive process, with which the spleen appears to have some obscure connexion. The degree of doubt and uncertainty which hangs over the functions of the spleen deprives its pathology of that interest which is attached to affections of other organs, whose functions and importance to the economy are better understood. We are of opinion that a catalogue of mere pathological changes, which have no bearing upon either physiology or therapeutics, is, in the present state of our knowledge, a matter of simple medical curiosity, or, as Dr. Bright elegantly says, "an alphabet for the construction of a language, into which we may *hereafter* translate the complicated and obscure legends of disease."

Dr. Bright's paper commences with some remarks upon the relative anatomy of the spleen; the author then gives a sketch of its general pathology; devotes a few considerations to the diagnosis of its diseases; and terminates with a detail of the cases. We see little worth noticing in the first part of the paper. After mentioning the situation of the spleen, and its general diversity of size, Dr. Bright informs us that, "great as this variety is, and great as is the occasional increase of this organ, it perhaps never, in its healthy state, descends below the margin of the ribs, or becomes sensible to the touch in that part of the abdomen."

The pathological changes chiefly noticed in the spleen are simple congestion; congestion with enlargement; fleshy hardness; fleshy hardness with enlargement; softening; inflammation; suppuration; gangrene; tubercles; malignant disease; melanosis; fibrinous deposits; bony deposits; cellular degeneration; hydatids; laceration; supernumerary spleens. The morbid conditions which Dr. Bright enumerates as proper to the peritoneal investment of the spleen are inflammation; ecchymosis; adhesions; fibrinous deposits; cartilaginous deposits; bony deposits; cicatrices; tubercles; and malignant deposits. This catalogue does not add anything to our knowledge on the subject of the pathology of the spleen: with the mere exception of tubercles and hydatids, it is pretty nearly the same account as that given by Portal, in 1803; since which period the subject of the acephalocyst of the spleen has been fully investigated by Cruveilhier. The local symptoms of certain diseases of the spleen consist chiefly in pain, tenderness on pressure, and distinct tumefaction; and such symptoms are common to those diseases of the spleen which consist in congestion, inflammation, or hypertrophy. It must be evident that a great number of the affections of this organ are not attended with symptoms of a character sufficiently marked to enable us to determine upon their nature during life; for example, abscesses of the spleen. Dr. Bright records two cases of abscess of this organ, bursting, one into the stomach and the other into the colon, in which nothing more than a conjectural diagnosis could be attempted. A great majority of cases of disease in this organ must necessarily be of the same character. The difficulties in diagnosis here principally arise from the situation of the spleen, the great obscurity hanging over its functions, and the degree of its importance to the economy at large. "The pain accompanying splenic disease is seldom acute, unless the peritoneal covering of the organ be inflamed; and then, owing to the proximity of so many other parts, as the heart, the lungs, the diaphragm, the stomach, the left kidney, and the colon, it is very difficult to localize; although, by the method of abstracting one by one of the organs, in proof of the lesions of which certain other symptoms are wanting, we may come to the conclusion that the pain belongs to the spleen."

Diseases of the spleen may be confounded with those of many other organs which are situated in its vicinity. These are chiefly "chronic abscess of the integuments; scirrhous thickening of the stomach; enlargement of the left lobe of the liver; diseased omentum; feculent accumulation in the colon; diseased kidney; ovarian dropsy; hydatids." The chronic abscess of the integuments is too superficial or too soft to be reasonably confounded with disease of an internal viscus, particularly one whose structure is so solid as the spleen. Some difficulty may arise in establishing a differential diagnosis between a scirrhoid thickening of the stomach and disease of the spleen. "In this case one of the best distinctive marks will be the sound elicited by percussion." The sound will be clear in thickening of the stomach, dull in disease of the spleen. Feculent accumulation in the intestines is very likely to be confounded with disease of the spleen, particularly when the accumulation exists in the descending colon and left portion of the arch. In these cases we must be guided by the history of the disease and the effects of remedies. "Nor must we, without the most persevering employ of purgatives, hastily conclude that the intestines have been emptied." Again, disease of the left kidney, in many of its features, simulates disease of the spleen. The chief distinctive marks are here the history of the disease, some probable changes in the properties of the urine, the more fixed tumefaction when the kidney is diseased, (it being situated further back than the splenic tumour would be,) and its not falling forwards when the patient is placed on his hands and knees. "On careful examination by percussion, also, we shall find there is reason to conclude that the intestine lies between the tumour and the integuments of the lower part of the abdomen when the kidney is enlarged."

Of the treatment of diseases of the spleen Dr. Bright says little. The frequency of the connexion of such states with affections of the economy generally, or with some portion of the digestive, absorbent, renal, or glandular systems, prevents us, in many instances, directing our treatment especially to the diseases of this organ, even when these are obvious. In such conditions, the treatment of the affection of the spleen becomes secondary to that of the general state of the economy, or any of its chief systems. In well-marked acute affections of this organ with enlargement, as in the first and second cases given by Dr. Bright, local bleedings, with purgatives and tonics, are indicated.

This paper concludes, for the present, the pathological contributions of Dr. Bright. It is not of so much interest as the two former; neither does it present as much novelty, not laying before us any new facts in the history of splenic diseases. Still it is of great merit: it gives us more facts, furnishes us with more data, and establishes a firmer and broader basis on which to build a correct diagnosis. We feel deeply indebted to Dr. Bright for these and former communications, and doubt not the profession at large will join with us in hoping that he may be enabled to proceed with his interesting enquiries, by the part of which already published he has laid his brethren throughout the world under important obligations.

---

